# Unlocking Antimicrobial Peptides from Marine Invertebrates: A Comprehensive Review of Antimicrobial Discovery

**DOI:** 10.3390/antibiotics14090924

**Published:** 2025-09-12

**Authors:** Md. Abu Kawsar, Chengqing Zhao, Fan Mao, Ziniu Yu, Yang Zhang

**Affiliations:** 1 State Key Laboratory of Breeding Biotechnology and Sustainable Aquaculture, Guangdong Provincial Key Laboratory of Applied Marine Biology, South China Sea Institute of Oceanology, Chinese Academy of Sciences, Guangzhou 510301, China; kawsar.aq@sau.ac.bd (M.A.K.); maofan@scsio.ac.cn (F.M.); carlzyu@scsio.ac.cn (Z.Y.); 2 University of Chinese Academy of Sciences, Beijing 100049, China; 3 Department of Aquaculture, Sylhet Agricultural University, Sylhet 3100, Bangladesh; 4 Sinopep Allsino Bio Pharmaceutical Co., Ltd., Lianyungang 222000, China; chengqing.zhao@sinopep.com

**Keywords:** marine invertebrates, antimicrobial peptides (AMPs), antimicrobial resistance (AMR), peptide therapeutics, artificial intelligence, drug discovery

## Abstract

Unlike other animals, marine invertebrates lack an adaptive immune system and instead rely on innate immunity as their first line of defense. A key component of this innate response is the production of biologically active molecules, particularly antimicrobial peptides (AMPs), which offer promising solutions to the escalating global crisis of antimicrobial resistance (AMR). This review comprehensively examines the sources, structural diversity, mechanisms of action, biological functions, and therapeutic potential of AMPs derived from a wide range of marine invertebrate phyla. These evolutionarily conserved peptides exhibit broad-spectrum antibacterial, antifungal, antiviral, antiparasitic, and even anticancer activities. The review also summarizes strategies for AMP isolation and production, ranging from natural extraction to recombinant expression and chemical synthesis, and outlines their potential biotechnological applications. Furthermore, we highlight the transformative role of artificial intelligence (AI) in accelerating AMP discovery, design, and production, including predictive modeling, de novo peptide generation, and optimization workflows. Despite significant progress, challenges remain in large-scale production, pharmacokinetic characterization, and functional validation. Addressing these gaps through integrative omics, structural biology, and AI-driven innovation will be crucial for unlocking the full therapeutic potential of marine invertebrate AMPs in combating infectious diseases and antimicrobial resistance.

## 1. Introduction

Marine ecosystems, covering more than 70% of the Earth’s surface, represent the largest ecosystem on the planet and are recognized as highly diverse and chemically distinctive environments [1,2,3]. The extreme and fluctuating conditions of these habitats, including high hydrostatic pressure, variable salinity, low temperatures, and limited light have driven the evolution of structurally unique and chemically diverse metabolites, many of which are absent in terrestrial organisms [4,5]. Marine microorganisms such as fungi, bacteria, myxomycetes, and microalgae are prolific producers of secondary metabolites with a wide range of bioactivities, including antioxidants, antimicrobial, antiviral, and anticancer properties [6,7]. Shaped by intense ecological pressures, these compounds have garnered significant interest for applications across food technology, pharmaceuticals, and biotechnology. Notably, marine-derived bioactive molecules such as peptides, polysaccharides, omega-3 fatty acids, and phenolic compounds have demonstrated therapeutic potential in managing oxidative stress, cardiovascular disease, metabolic disorders, and infectious diseases [7]. Over the past few decades, many of these compounds have progressed into pharmacological development, with peptides gaining particular importance due to their structural specificity, functional versatility, and broad-spectrum antimicrobial efficacy [8,9].

Marine peptides’ short protein fragments exert biological functions beyond nutrition, including antimicrobial, antioxidant, anticoagulant, and immunomodulatory effects [4,5,9]. Isolated from diverse marine taxa such as algae, mollusks, crustaceans, fish, bacteria, and fungi, their bioactivity is tightly linked to amino acid sequences, structural motifs, and physicochemical properties [10]. Among these, antimicrobial peptides (AMPs) are evolutionarily conserved effectors of innate immunity in both invertebrates and vertebrates [8]. They act as rapid responders to microbial invasion, primarily by interacting with and disrupting bacterial membranes, causing cell lysis [8,11]. AMPs are widely distributed in epithelial tissues, phagocytes, and bodily fluids, where they can be constitutively expressed or induced upon microbial challenge or inflammation [12]. Beyond extracellular activity, AMPs are critical for intracellular pathogen clearance within phagocytes and contribute to maintaining mucosal defense [11]. Their biosynthesis typically involves proteolytic cleavage from precursor proteins, with dysregulation implicated in inflammatory diseases such as Crohn’s disease and atopic dermatitis [11]. Furthermore, many AMPs modulate immunity by regulating cytokine expression, enhancing chemotaxis, and promoting tissue repair [13].

Recent advances have revealed that AMPs possess functions beyond immune defense. In *Drosophila melanogaster*, CRISPR/Cas9-mediated studies demonstrate that individual AMPs have specific, non-redundant roles in immunity [14]. Additionally, some AMPs participate in neurobiological processes, including sleep regulation, memory formation, and neuroprotection. Dysregulation of AMP expression correlates with neurological disorders such as Alzheimer’s disease, Parkinson’s disease, and frontotemporal dementia [14]. These findings highlight AMPs as multifunctional biomolecules that not only provide frontline antimicrobial defense [11,15] but also influence broader physiological and immunological processes [13,14]. Their evolutionary resilience, structural diversity, and broad bioactivity underscore their potential as scaffolds for next-generation therapeutics targeting infection, inflammation, and immune dysfunction [11,16].

While considerable progress has been made, the escalating threat of antimicrobial resistance (AMR) continues to pose a critical challenge to global public health. The widespread and indiscriminate use of antibiotics in human medicine, agriculture, and aquaculture has driven the emergence and proliferation of multidrug-resistant (MDR) pathogens [17]. The World Health Organization has classified MDR organisms as a major global health concern requiring urgent intervention [18]. Alarmingly, antibiotic development has not kept pace with resistance evolution; between 1960 and 2010, only four new antibiotic classes were introduced, most of which are derivatives of existing scaffolds [19]. In response to the diminishing efficacy of conventional antibiotics, marine-derived antimicrobial peptides (AMPs), particularly those from microalgae, have gained increasing attention as promising alternatives. These peptides exhibit broad-spectrum antimicrobial activity, a low propensity for resistance development, and minimal cytotoxicity [20]. Notably, microalgal AMPs have demonstrated potent activity against clinically and aquaculturally relevant MDR pathogens, including *Staphylococcus aureus*, *Pseudomonas aeruginosa*, and *Vibrio* species [21,22]. Unlike traditional antibiotics, AMPs primarily target and disrupt bacterial membranes, a mechanism that is inherently less prone to resistance development [23]. Given the disproportionate impact of infectious diseases and AMR in low- and middle-income countries, marine AMPs offer sustainable, biocompatible, and potentially transformative alternatives for both human and veterinary medicine [9,24,25].

Driven by technological innovation and clinical necessity, marine AMP research is rapidly evolving. High-throughput sequencing and omics approaches such as transcriptomics and proteomics have accelerated discovery of novel AMPs across diverse taxa [26]. More recently, artificial intelligence (AI) and machine learning (ML) are revolutionizing AMP discovery and design, enabling activity prediction, rational optimization, and 3D structure modeling that expedite translation from sequence to functional application [27]. This review synthesizes current knowledge on marine antibacterial peptides, focusing on their sources, mechanisms of action, and therapeutic applications. We further discuss advances in AI-driven peptide discovery alongside challenges and future directions for translating marine peptides into clinically and industrially viable antimicrobial agents. Although several reviews have addressed marine natural products or the therapeutic potential of antimicrobial peptides in general, few have provided a focused and integrative synthesis on peptides derived specifically from marine invertebrates. This review provides comprehensive coverage of the structural and functional diversity of marine invertebrate AMPs, their mechanisms of antimicrobial action and immunomodulatory roles, and the integration of advances in AI- and omics-driven discovery pipelines that link traditional biochemical characterization with modern computational strategies. By combining classical perspectives with emerging technologies, it offers a consolidated framework that highlights both current knowledge and future opportunities in marine AMP-based drug discovery.

## 2. Marine Invertebrate-Based Antimicrobial Peptides

Marine organisms represent a prolific source of antimicrobial peptides (AMPs), shaped by the unique ecological challenges of their aquatic environments. Unlike terrestrial organisms, many marine species, particularly invertebrates lack adaptive immune systems and thus rely primarily on innate defenses, including AMPs, to counteract bacterial, fungal, and viral threats [28]. The high microbial load in seawater, coupled with the constant risk of biofouling and infection, has exerted strong selective pressure for the evolution of structurally diverse and functionally potent AMPs [29]. These peptides, commonly known as host defense peptides or alarmins, represent a vital frontline element of innate immunity in numerous marine invertebrates, with the hemolymph being a principal site of their production [30]. To date, nearly 3000 antimicrobial peptides (AMPs) have been discovered across a diverse array of organisms, including plants, mammals, aquatic vertebrates, and invertebrates, underscoring their evolutionary conservation and essential function in host defense [30].

**Figure 1:**
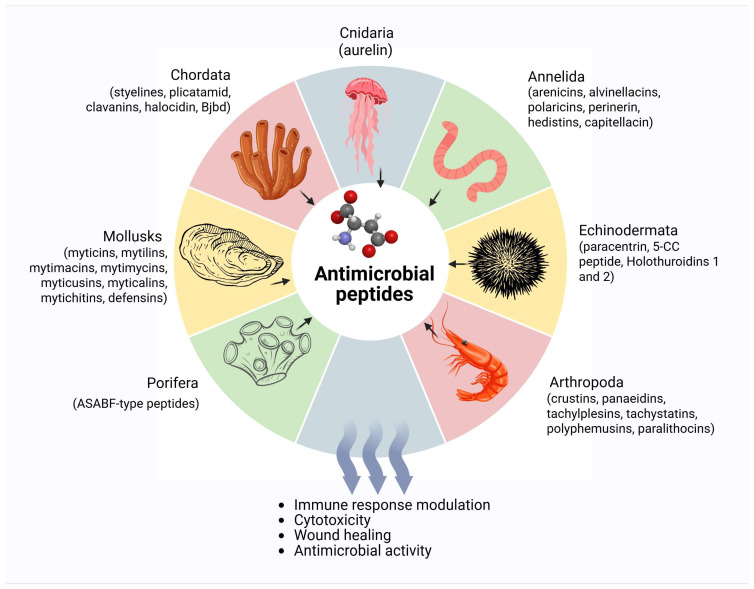
Major sources and diverse biological functions of antimicrobial peptides (AMPs) derived from marine invertebrates.

The structural diversity of marine AMPs including α-helical, β-sheet, cysteine-rich, and cyclic peptides reflects their adaptive responses to diverse and evolving microbial threats. These structural classes often feature specialized modifications such as cyclization, halogenation, and disulfide bonding, which enhance antimicrobial efficacy and stability in saline marine conditions [26,30]. Figure 1 illustrates the diverse marine invertebrate sources of antimicrobial peptides (AMPs) and highlights their wide-ranging biological activities. Additionally, marine species are frequently exposed to environmental fluctuations in salinity, temperature, and pathogen pressure, necessitating a robust and flexible antimicrobial arsenal. Despite extensive research, the marine biosphere remains largely underexplored, suggesting that many AMPs with novel structures and mechanisms of action await discovery. This ecological and evolutionary richness positions marine environments as a promising frontier for uncovering next-generation antimicrobial agents.

**Figure 2:**
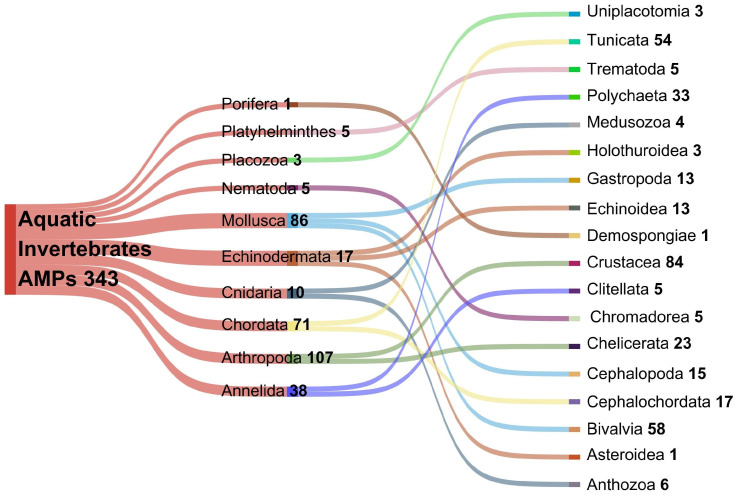
Distribution of antimicrobial peptides (AMPs) among aquatic invertebrate phyla and their respective classes.

Aquatic invertebrates, in particular, represent some of the most ancient and globally distributed metazoan lineages and have developed highly specialized immune strategies to combat microbial threats in both stable and human-impacted ecosystems [31]. Historically thought to possess only innate immunity, invertebrates mount cellular and humoral responses mediated by pattern recognition receptors [32]. Notably, recent evidence supports the presence of immunological priming in some invertebrate species, suggesting a form of immune memory with prolonged protective effects, although the underlying molecular pathways remain poorly characterized [33]. The cellular immune component is driven by hemocytes in the hemolymph, facilitating phagocytosis, encapsulation, and nodule formation. Concurrently, the humoral response is mediated through coagulation cascades and the release of antimicrobial compounds, with AMPs playing a central role [34]. These peptides disrupt microbial membranes through electrostatic and hydrophobic interactions, a mechanism enhanced in marine invertebrates by their adaptation to high-salinity environments resulting in greater stability and broader-spectrum antimicrobial activity [35,36,37]. With over 40 AMP families characterized, aquatic invertebrates remain the principal source of marine AMPs, offering a deep and underutilized reservoir for antimicrobial discovery [38]. The current review highlights this diversity, organizing AMP data phylum-by-phylum to showcase the structural and functional versatility of invertebrate-derived peptides (Figure 2).

## 3. Marine Phyla Producing AMPs

### 3.1. Annelida

Annelids constitute a phylogenetically important group of primitive coelomates within the Lophotrochozoa, distinguished by their broad ecological distribution and complex immune capabilities [39]. Inhabiting both freshwater and marine environments including extreme habitats such as hydrothermal vents, polar regions, and contaminated sediments annelids have evolved highly effective defense mechanisms to thrive in microbe-dense ecosystems, making them valuable models for the study of bioactive molecules [40]. Their immune system is primarily driven by cellular responses, including phagocytosis, encapsulation, and coelomocyte-mediated cytotoxicity, and is complemented by a well-developed humoral arm involving hemolytic, coagulation, and antimicrobial components circulating in the body fluid [39,41]. Although antimicrobial peptide (AMP) research has historically focused on arthropods, annelids offer unique insights due to their evolutionary position and distinct immunological architecture. Recent research has identified 38 antimicrobial peptides (AMPs) from 14 annelid species, categorized into six distinct peptide families. These AMPs exhibit broad-spectrum activity against a variety of pathogens, including *Candida albicans*, Gram-positive, and Gram-negative bacteria—many of which are particularly important in aquaculture (Table 1) [42]. Together, these findings highlight the considerable, yet largely unexplored, potential of annelids as a valuable reservoir of structurally diverse AMPs with promising applications in therapeutic development and microbial control.

### 3.2. Arthopoda

Arthropods constitute the most diverse and ecologically dominant phylum in the animal kingdom, comprising chelicerates, crustaceans, arachnids, and myriapods [12]. Although new arthropod species continue to be found, the number of known aquatic species remains relatively modest. Estimates suggest that approximately 100,000 to 110,000 aquatic arthropod species exist, including nearly 100,000 aquatic insects spanning 12 orders—together accounting for about 60% of all known aquatic animal species [59,60]. This remarkable diversity is driven by a combination of ecological adaptability and evolutionary innovation.

A major contributor to their adaptability is their highly developed innate immune system, which compensates for the absence of adaptive immunity. Arthropods rely on pattern recognition receptors (PRRs) and downstream effector molecules particularly antimicrobial peptides (AMPs), to detect and neutralize invading pathogens. A recent study identified 107 AMPs in aquatic arthropods, distributed across two primary classes: Chelicerata (23 AMPs) and Crustacea (84 AMPs) (Figure 1) [12]. These AMPs demonstrated antimicrobial activity against 86 microbial species, including 47 pathogens of particular concern in aquaculture: 13 Gram-positive bacteria, 25 Gram-negative bacteria, eight fungi, and the White Spot Syndrome Virus (WSSV). These results emphasize the immunological importance of arthropod-derived AMPs and their promising potential for the development of sustainable antimicrobial approaches in aquaculture.

#### Crustacea

Crustaceans, one of the most diverse marine arthropod classes, possess a robust innate immune system in which AMPs play critical roles. Several key examples of crustacean AMPs, along with their structures, sources, and antimicrobial activities, are listed in Table 2. These peptides are primarily expressed in hemolymph and epithelial tissues, where they contribute to the detection and elimination of microbial invaders [61]. Most crustacean AMPs are gene-encoded, cationic molecules synthesized by hemocytes-circulating immune cells in the open circulatory system. Some AMPs are proteolytically derived from precursor proteins originally associated with unrelated physiological functions, reflecting the evolutionary plasticity of crustacean immunity [61].

To date, 15 distinct AMP families have been characterized in crustaceans, with 14 of them reported in decapods such as shrimp, crabs, and lobsters. Among these, penaeid shrimp have received the most attention due to their high economic value in global aquaculture [62]. However, the intensive farming of shrimp is often undermined by recurring disease outbreaks caused by bacterial and viral pathogens. This challenge has spurred targeted research into shrimp immune mechanisms—particularly AMP-mediated defenses—as a foundation for innovative disease prevention strategies [62]. Beyond shrimp, AMPs have also been identified in a wide range of decapods, including crabs, lobsters, freshwater prawns, and crayfish, as well as in terrestrial crustaceans such as isopods, underscoring their evolutionary and functional diversity.

**Figure 3:**
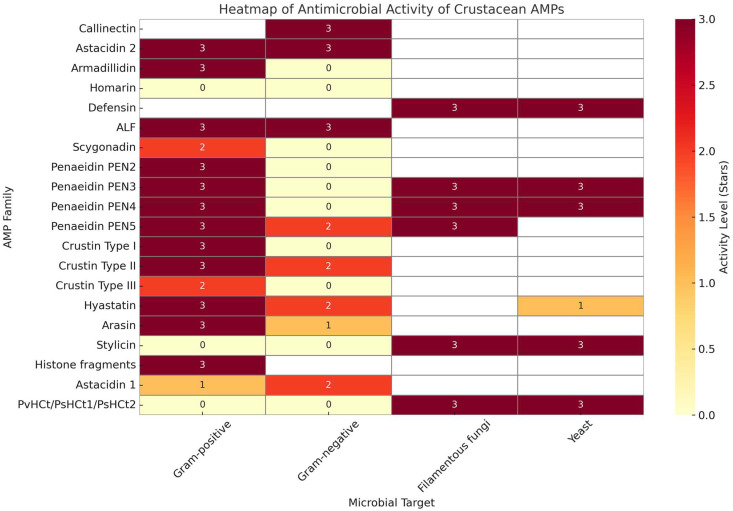
Heatmap of antimicrobial activity of crustacean antimicrobial peptides (AMPs) against different microbial targets. Note: Color intensity represents relative activity levels based on minimal inhibitory concentration (MIC) values: 3 (strong, ≤10 μM), 2 (moderate, 10–20 μM), 1 (weak, 20–40 μM), and 0 (inactive, >40 μM). Blank cells indicate AMP–microbe combinations that have not been tested or for which no data are available in the literature.

Figure 3 presents a heatmap illustrating the antimicrobial spectrum of various crustacean-derived AMPs, based on minimal inhibitory concentration (MIC) values. The intensity of the color gradient reflects relative potency against different microbial targets, including Gram-positive and Gram-negative bacteria, filamentous fungi, and yeast. Among the AMP families, anti-lipopolysaccharide factors (ALFs) exhibit broad-spectrum activity and maintain antimicrobial efficacy under high-salinity conditions, making them particularly effective in marine environments [63,64]. Crustins—especially Types I and II demonstrate strong antibacterial activity, with some variants requiring elevated salt concentrations to function optimally [65]. In contrast, certain penaeidins lose activity under saline conditions, as demonstrated for PEN3-1 [64]. Notably, the heatmap also highlights substantial data gaps for several AMP families, indicating limited antimicrobial screening and functional characterization, particularly in non-decapod crustaceans. These limitations underscore the need for broader microbial assays and validation under physiologically relevant conditions to evaluate the therapeutic potential of these AMPs [66,67].

**Figure 4:**
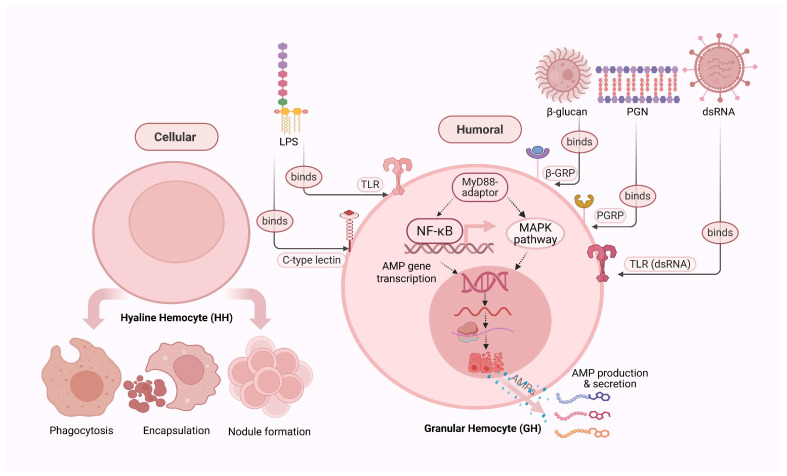
Schematic overview of crustacean immune responses following microbial challenge. Recognition of pathogen-associated molecular patterns (PAMPs) including lipopolysaccharides (LPS), peptidoglycans (PGN), β-glucans, and double-stranded RNA (dsRNA) occurs via membrane-bound and soluble pattern recognition receptors (PRRs) such as Toll-like receptors (TLRs), peptidoglycan recognition proteins (PGRPs), β-glucan-binding proteins (β-GRPs), and C-type lectins. LPS and PGN primarily bind TLRs and PGRPs, β-glucan is detected by β-GRPs, while dsRNA is sensed by TLR-like receptors. These interactions activate intracellular signaling cascades, including the NF-κB and MAPK pathways, leading to AMP gene transcription and peptide secretion by granular hemocytes (GH). Concurrently, hyaline hemocytes (HH) mediate phagocytosis, encapsulation, and nodule formation. Together, these cellular and humoral mechanisms reinforce the coordinated innate immune response of crustaceans.

Although multiple AMP families and isoforms have been identified in crustaceans—especially decapods—their concurrent expression in hemocytes suggests that they may act cooperatively to bolster immune defenses [68]. While direct evidence of synergy is limited, and no synergistic activity was observed between Litvan PEN2-1 and PEN3-1 in *Litopenaeus vannamei*, the possibility of functional cooperation remains plausible. In addition to AMPs, crustacean hemocytes produce other key immune effectors, including lysozyme and enzymes of the prophenoloxidase (proPO) cascade. Lysozyme, in particular, may synergize with AMPs by degrading bacterial peptidoglycan, thereby facilitating peptide access to bacterial membranes [69]. As illustrated in Figure 4, this intricate interplay between AMPs and other immune molecules highlights the multifaceted and highly coordinated nature of crustacean innate immunity.

**Table 2:** Representative Antimicrobial Peptides (AMPs) from Chelicerata and Crustacea.

Group	Peptide Name	Source Organism	Structure/Class	Key Features	Antimicrobial Activity	Reference(s)
Chelicerata	Tachyplesin	*T. tridentatus*, *C. rotundicauda*, *T. gigas*	Short cationic peptide, 2 disulfide bonds	Cyclized forms, enhanced stability	Bacteria, fungi, cancer cells, biofilms	[52,56,58,70,71,72,73,74]
	Polyphemusin	*Limulus polyphemus*	Tachyplesin-like structure	Amphipathic variants, high activity	Broad-spectrum	[52,58,71,75]
	Tachycitin	*T. tridentatus*	αβ-motif, chitin-binding, 10 cysteines	Synergistic with big defensin	Broad-spectrum	[76,77]
	Tachystatin	*T. tridentatus*, *T. gigas*	β-sheet, chitin-binding, 3 disulfide bonds	Isoform C: amphiphilic and hemolytic; A2: stable, non-toxic	Broad-spectrum	[78,79,80,81,82,83,84]
	Tatritin	*T. tridentatus*	α-helix + β-sheet, 6 disulfide bonds	Chitin-binding, stable structure	Broad-spectrum	[82,85,86]
Crustacea	Arasin	*C. sapidus*, various crabs, crayfish, prawns	Pro/Arg-rich N-terminal, Cys-rich C-terminal	Chitin-binding; immune regulation	Broad-spectrum	[30,65,87,88,89,90,91,92,93,94,95,96,97,98,99]
	Crustin	*C. maenas*, penaeid shrimp	WAP-domain-based peptide	7 structural types (I–VII); diverse bioactivities	Gram ± bacteria, fungi, viruses	[65,66]
	Proline-rich AMP	*C. maenas*, *S. paramamosain*	6.5 kDa, proline-rich	Non-lytic, blocks protein synthesis	Broad-spectrum	[32,36,100,101,102,103]
	Glycine-rich AMP	*S. paramamosain*	Gly-rich motifs, Cys-terminal	Thermally stable	Broad-spectrum	[89,104,105,106]
	Hyastatin	*Hyas araneus*	Multi-domain, chitin-binding, 6 Cys	Highly diverse; 14 variants in *P. trituberculatus*	Bacteria, chitin-binding	[107,108,109,110,111]

### 3.3. Chordata

Invertebrate members of the phylum Chordata, including Cephalochordates and Tunicates, remain relatively underexplored in the field of antimicrobial peptide (AMP) discovery. Although small in number, a total of 71 AMPs from 11 distinct families have been identified from seven invertebrate chordate species comprising one amphioxus (Cephalochordata) and six ascidians (Tunicata) [58]. These peptides exhibit antimicrobial activity against 40 microbial species, notably including 26 pathogens relevant to aquaculture: eight Gram-positive bacteria, 14 Gram-negative bacteria, and four fungi [12]. These findings underscore the significant potential of invertebrate chordates, particularly ascidians, as promising sources of novel bioactive compounds for combating pathogens in aquatic environments (Table 3).

### 3.4. Cnidaria

Cnidarians are an ancient and taxonomically rich phylum composed primarily of marine organisms, characterized by specialized stinging organelles called cnidocytes used for defense and prey capture [130,131]. They are divided into three major clades Anthozoa, Medusozoa, and Myxozoa, of which Anthozoa and Medusozoa are best represented in AMP studies [132,133]. Although they lack adaptive immunity, cnidarians exhibit complex innate immune responses, including immune recognition, signaling cascades, and effector mechanisms [134,135]. A recent analysis identified 10 antimicrobial peptides (AMPs) representing 10 distinct families from eight cnidarian species six from Anthozoa and two from Medusozoa demonstrating activity against 27 microbial species [136]. Notably, 22 of these microbes are pathogens relevant to aquaculture, including six Gram-positive and 16 Gram-negative bacteria [12]. These results underscore the immunological diversity of cnidarians and highlight their largely untapped potential as sources of structurally diverse, broad-spectrum AMPs for controlling aquatic pathogens and developing novel antimicrobial agents (Table 4).

### 3.5. Echinodermata

The phylum Echinodermata includes over 7500 exclusively marine invertebrate species, classified into five major classes: Asteroidea, Crinoidea, Echinoidea, Holothuroidea, and Ophiuroidea [148,149]. These organisms rely on an innate immune system mediated by coelomic fluid, which contains coelomocytes immune effector cells responsible for producing antimicrobial agents such as lysozymes and antimicrobial peptides (AMPs) [150]. Although genomic studies have revealed a surprisingly complex repertoire of immune-related genes in echinoderms, the functional understanding of their immune mechanisms remains limited [151]. Recent investigations have identified 17 AMPs from six echinoderm species—specifically, one *Asteroidea*, four *Echinoidea*, and one *Holothuroidea* distributed across five AMP families. These peptides have demonstrated antimicrobial activity against 20 microbial species, including 13 aquaculture-relevant pathogens: five Gram-positive bacteria, six Gram-negative bacteria, and two fungal species [152]. The identified echinoderm-derived AMPs and their key features are summarized in Table 5. 

### 3.6. Mollusca

Mollusks, the second-largest invertebrate phylum with over 50,000 species across eight classes, have emerged as a prolific source of antimicrobial peptides (AMPs), particularly from Bivalvia, Cephalopoda, and Gastropoda, which together account for more than 95% of molluscan AMP diversity [58]. To date, 86 AMPs belonging to 17 families have been identified from 28 molluscan species, exhibiting activity against 71 microbial species, including critical aquaculture pathogens, i.e., *Staphylococcus aureus* and *Vibrio* spp. [159]. Among bivalves, mussels and oysters produce cysteine-rich AMPs such as mytilins, myticins, mytimycins, and myticusins with broad-spectrum antibacterial, antifungal, antiviral, and antiparasitic activity (Table 6) [95,160,161]. Additional structurally diverse bivalve peptides, including myticalins, mytichitins, Ap, Cg-Prp, and URP20, further expand their functional repertoire [47,162,163]. Cephalopods contribute AMPs such as octopartenopin, octominins, and peptides from *Sepia officinalis*, which have demonstrated potent antibacterial properties [99,164,165,166]. In gastropods, AMPs like Cm-p1, Pom-1, Pom-2, and Dolabellanin B2 exhibit antibacterial, antifungal, and antiviral activities, often with low cytotoxicity, underscoring their therapeutic promise [167,168,169,170]. Collectively, the structural and functional diversity of molluscan AMPs highlights their potential as valuable candidates for developing novel antimicrobial strategies in aquaculture and biomedicine.

### 3.7. Nematoda

The phylum Nematoda, comprising over 28,000 described species across the classes *Chromadorea* and *Enoplea* [53,164], remains largely underexplored in terms of antimicrobial peptide (AMP) diversity, particularly among aquatic species [12]. While terrestrial nematodes such as *Caenorhabditis elegans* have been extensively studied for their innate immune effectors, information on AMPs in aquatic nematodes remains limited [187]. A notable exception is the discovery of anisaxins a group of cecropin-like, α-helical AMPs isolated from the marine parasitic nematode *Anisakis*, a known causative agent of anisakiasis. These peptides exhibit potent bactericidal activity against Gram-negative bacteria while maintaining low cytotoxicity toward human cells [188]. More recently, a computational pipeline combining homology-based prediction and structural screening was used to identify novel helminth-derived AMPs from both nematodes and platyhelminths. This approach led to the synthesis of several candidate peptides, among which four- nAMP-LP-18, nAMP-LP-104, nAMP-LP-249, and nAMP-LP-298 demonstrated measurable antibacterial activity (minimum inhibitory concentration, MIC < 100 µg/mL) against at least one tested bacterial strain [189].

### 3.8. Placozoa

Marine Placozoa, one of the most ancient and structurally simple animal phyla, have recently gained attention as a promising source of antimicrobial peptides (AMPs). These flat, amoeba-like invertebrates inhabit shallow tropical and subtropical seas, where they constantly interact with diverse microbial communities on submerged surfaces. Despite their lack of specialized tissues, genomic and transcriptomic analyses of *Trichoplax adhaerens* the most studied placozoan have revealed genes encoding putative AMPs, including cysteine-rich peptides, β-defensin-like molecules, and a unique family known as Trichoplaxins [190]. Trichoplaxins are α-helical peptides known for their potent antimicrobial activity against both bacteria and fungi. Notably, a synthetic analog, Trichoplaxin-2A, exhibits strong and selective anticancer activity while maintaining low cytotoxicity toward mammalian cells [190,191]. Although only four species within the phylum Placozoa have been formally described, phylogenomic and gene content analyses suggest that this group harbors significantly greater hidden diversity [127,192]. Given their basal position in the metazoan lineage and unique epithelial organization, AMPs from Placozoa may represent some of the earliest and most structurally novel immune effectors [193]. Continued exploration of placozoan peptides holds great potential not only for understanding the evolution of innate immunity but also for discovering new scaffolds for antimicrobial and anticancer drug development.

### 3.9. Platyhelminthes

The phylum *Platyhelminthes*, encompassing both parasitic and free-living flatworms such as *Schistosoma mansoni* and various marine turbellarians remains relatively understudied in the context of antimicrobial peptide (AMP) discovery. While parasitic species like *S. mansoni*, the causative agent of schistosomiasis affecting millions globally, have received more extensive attention [58,194], AMP research in free-living marine flatworms is still in its early stages [80,189]. A notable example is SmDLP, an AMP identified from *S. mansoni*, which exhibits structural similarity to dermaseptin 3.1 from the frog *Agalychnis annae*. SmDLP displays potent antimicrobial and hemolytic activity, suggesting a potential role in host immune evasion and parasite survival [82]. Computational analyses have revealed that flatworms typically lack canonical AMP families found in other taxa. Instead, they possess a repertoire of structurally distinct, often cysteine-rich AMP-like peptides. Recently, two such peptides—fAMP-LP-5 and fAMP-LP-17 were identified and shown to exhibit antibacterial activity at concentrations below 100 µg/mL, underscoring their potential as novel candidates for antimicrobial drug development [189].

In contrast to the growing AMP research in other marine invertebrates such as polychaetes, mollusks, cnidarians, and tunicates which has uncovered structurally diverse AMPs like β-hairpin arenicins, BRICHOS-domain peptides, and defensin-like families [12], marine flatworms remain largely unexplored. The scarcity of AMP reports from marine Platyhelminthes likely reflects a lack of comprehensive genomic and transcriptomic studies in this group. However, given their constant exposure to rich microbial communities in coral reef, intertidal, and benthic environments, it is plausible that marine flatworms harbor novel AMPs with distinct structural and functional features. Future research incorporating high-throughput transcriptomics and functional peptide screening may uncover new AMP scaffolds from marine flatworms, contributing to the expanding landscape of marine-derived antimicrobial drug discovery.

### 3.10. Marine Porifera-Derived Antimicrobial Peptides

Sponges (*Phylum porifera*), regarded as the most ancient and primitive extant metazoans, are recognized as prolific producers of structurally diverse bioactive compounds, including antimicrobial peptides (AMPs). Of the more than 9300 described sponge species over 80% of which belong to the class *Demospongiae* many engage in complex symbiotic associations with microbial communities. These microbial symbionts are now believed to be responsible for producing a substantial portion of the chemical compounds historically attributed to the sponges themselves [195]. Between 1990 and 2019, sponges accounted for nearly half of all marine natural products identified from invertebrates, underscoring their exceptional chemical diversity and biotechnological potential [196,197]. Notably, sponge-derived AMPs exhibit broad-spectrum bioactivity, including antibacterial, antifungal, antiviral, and antifouling properties [2,4,16].

One of the few well-characterized antimicrobial peptides (AMPs) from sponges is ASABF_SUBDO, a 64-amino-acid defensin-like peptide isolated from *Suberites domuncula*. Structurally defined by a cysteine-stabilized αβ (CSαβ) fold, ASABF_SUBDO shares high sequence similarity with ASABF peptides previously identified in the terrestrial nematode *Ascaris suum*, and exhibits potent antimicrobial activity against both bacteria and fungi, as well as hemolytic activity toward erythrocytes [4,198]. Additional sponge-derived AMPs include a variety of cyclic peptides and depsipeptides. Notable examples are Discodermins A–D from *Discodermia* spp., which exhibit strong antibacterial activity, and Halicylindramides A–E from *Halichondria* spp., recognized for their antifungal properties [199]. Furthermore, compounds such as theonellamide F and its analogs, isolated from *Theonella swinhoei*, have demonstrated significant antifungal activity. Antiviral peptides—including Mirabamides and Stellettapeptins—have also been identified, with both classes showing inhibitory effects against HIV [29].

Together, these findings underscore the extraordinary chemical and structural diversity of sponge-derived AMPs including linear cysteine-rich peptides, β-hairpin motifs, and macrocyclic scaffolds which reflect the evolutionary adaptation of sponges to microbe-rich marine environments. As such, marine Porifera remain a critical reservoir for the discovery of novel peptide-based therapeutics with wide-ranging biomedical applications [200,201,202]. The major bioactive peptides derived from marine Porifera and their reported activities are summarized in Table 7.

## 4. Isolation and Production of Marine-Derived AMPs

**Figure 5:**
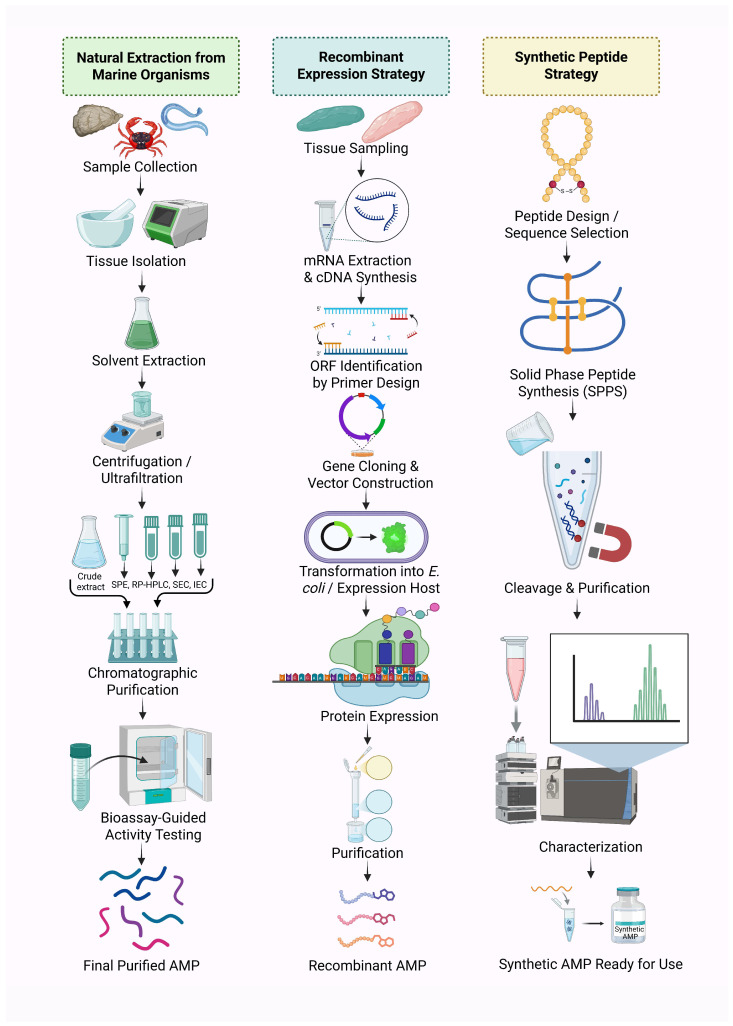
Schematic representation of three major strategies for the isolation and production of AMPs from marine sources. Natural extraction involves solvent-based recovery and chromatographic purification from marine organisms, followed by bioassay-guided activity testing. The recombinant expression strategy includes mRNA extraction, open reading frame (ORF) identification, gene cloning, heterologous expression, and purification. Synthetic peptide production utilizes solid-phase peptide synthesis (SPPS), followed by cleavage, purification, and analytical characterization. These complementary approaches enable the discovery and scalable production of bioactive AMPs for therapeutic applications.

The recovery of antimicrobial peptides (AMPs) from marine organisms is inherently complex due to several influencing factors, including habitat variability, seasonal abundance, and species-specific ecological traits, all of which impact the availability and yield of bioactive compounds [208,209,210]. The discovery pipeline typically begins with the collection of marine organisms and preliminary bioactivity screening targeting antibacterial, antifungal, or antiviral properties. This is followed by tissue dissection, mechanical disruption, and solvent-based extraction. Initial purification steps, such as centrifugation, precipitation, and membrane filtration, are employed to eliminate cellular debris and concentrate peptide-rich fractions [29]. Subsequently, advanced chromatographic techniques—solid-phase extraction (SPE), reversed-phase high-performance liquid chromatography (RP-HPLC), size-exclusion chromatography (SEC), and ion-exchange chromatography (IEC)—are utilized to isolate bioactive peptides and remove non-peptidic contaminants such as fatty acids and salts [65,211]. These three primary strategies-natural extraction, recombinant production, and chemical synthesis are illustrated in Figure 5.

Multiple marine AMPs have been successfully isolated using these strategies. For instance, Mytilus-derived defensins, mytilins, and mytimycins were purified from hemocytes using a combination of acetic acid extraction, C18-SPE, RP-HPLC, SEC, and additional rounds of RP-HPLC [174,209]. Similarly, perinerin from clamworm was isolated through acid extraction, followed by heparin-affinity chromatography and RP-HPLC [74], while crustin (carcinin) from shore crab hemocytes was obtained using ion-exchange chromatography and SEC after acetic acid extraction and dialysis [212]. Arenicins from lugworm coelomocytes were extracted using ultrafiltration and further purified by RP-HPLC following acidic treatment [213]. This multistep purification approach, often guided by iterative bioassays, ensures the retention of functionally validated peptides—a method commonly referred to as bioassay-guided purification [65,212] (Figure 5).

To meet the growing demand for antimicrobial peptides (AMPs) in basic research, therapeutics, and clinical trials, alternative production strategies have been developed to overcome the limitations of natural sourcing [46,214]. Direct isolation from organisms and chemical synthesis yield only small amounts, are costly, and often produce peptides with poor pharmacokinetics, low efficacy, and limited scalability [8,214]. One biological approach involves stimulating AMP expression in host organisms through pathogen exposure [215]. More notably, recombinant expression systems enable gene-based AMP production, offering a relatively low-cost, scalable alternative [216]. For example, CiHep, a hepcidin peptide from *Ctenopharyngodon idella*, was successfully expressed in *E. coli* and purified using affinity chromatography following mRNA extraction and cDNA synthesis [215,217]. *E. coli* and yeast are the primary hosts for recombinant AMP production, accounting for over 95% of reported cases [218]. While some AMPs achieve satisfactory yields in yeast [219,220], others are produced only in negligible amounts or inactive forms [216]. Despite these advances, challenges remain: disulfide-rich peptides often fold inefficiently, and carrier proteins (e.g., GST, thioredoxin, SUMO) can improve solubility but suffer from low yields, inefficient cleavage, or self-cleavage. AMP toxicity to microbial hosts further restricts production [216], and the absence of AMP-specific expression systems contributes to high costs and low scalability [216]. Recent studies have begun to address these limitations. For example, Epinecidin-1 (Epi-1) from the marine grouper (*Epinephelus coioides*) has been produced through rational peptide design, fusion expression, and enzymatic cleavage strategies, which enhance production efficiency, solubility, stability, and functional activity [221].

Chemical synthesis offers a scalable and reliable approach for producing antimicrobial peptides (AMPs), particularly when natural sources are limited or difficult to access. This method has been successfully applied to synthesize marine peptides such as ALFPm11 an anti-lipopolysaccharide factor from *Penaeus monodon* as well as cyclic peptides from *Stylissa carteri*, utilizing solid-phase and hybrid solution-phase synthesis techniques [222]. Recent advances in omics technologies including transcriptomics, proteomics, and metabolomics combined with high-resolution analytical platforms such as LC-MS/MS and integrated bioinformatics pipelines, have significantly accelerated the discovery, characterization, and validation of novel AMPs from marine organisms [223,224].

## 5. Potential Biotechnological and Therapeutic Applications

### 5.1. Use in Human and Veterinary Medicine

The rising global threat of antimicrobial resistance has intensified the search for alternative therapeutics with broad-spectrum efficacy and minimal toxicity. Among these, antimicrobial peptides (AMPs) have attracted significant attention due to their evolutionary conservation, diverse mechanisms of action, and effectiveness against a wide array of pathogens. Their versatile potential spans both human and veterinary medicine, offering a promising platform for innovative anti-infective therapies.

Particularly notable are food-derived AMPs, which possess translational advantages owing to their natural origin and well-established safety profiles. A prominent example is lactoferrin (Lf), an iron-binding glycoprotein derived from whey, which plays a crucial role in innate immunity. Lactoferrin and its proteolytic fragments—such as Lf(1–11), lactoferrampin, and notably lactoferricin (Lfcin) demonstrate enhanced antimicrobial potency compared to the full-length protein [225]. Among these, Lfcin is distinguished by its broad-spectrum activity against bacterial, fungal, and parasitic infections, positioning it as a strong candidate for therapeutic applications across a range of conditions, including ocular, osteo-articular, gastrointestinal, and dermatological diseases [225].

In addition to mucosal and systemic infections, AMPs are also being explored for their potential in treating bone infections, a domain where conventional antibiotics often fall short. In osteomyelitis, particularly after surgical debridement, residual microbial biofilms severely limit antibiotic efficacy by impeding drug penetration [226]. Moreover, the prolonged local administration of high-dose antibiotics can result in host cytotoxicity, bone tissue damage, and the emergence of resistant bacterial strains factors that significantly compromise healing [227,228]. These challenges underscore the need for biocompatible alternatives that combine safety with potent antimicrobial effects.

AMPs have shown considerable promise in this regard. For example, PL-5, a peptide-based topical spray, has demonstrated efficacy in treating skin and wound infections caused by drug-resistant pathogens, with clinical trials confirming its safety and therapeutic potential [226,229]. Likewise, hLF1-11, a synthetic N-terminal peptide derived from human lactoferrin, has shown effective antibacterial action in osteomyelitis animal models and has successfully progressed to phase I clinical trials, reinforcing its potential as a novel therapeutic for bone infection management [230]. In veterinary medicine, Lfcin and other lactoferrin-derived peptides have also demonstrated broad therapeutic applicability, particularly in livestock and companion animals. Notable applications include the treatment and prevention of bovine mastitis, control of enterotoxigenic *E. coli* infections in piglets, and management of otitis in dogs. Delivery strategies range from intramammary infusion and oral supplementation to recombinant peptide expression in milk or yeast systems. These interventions have shown significant outcomes in reducing pathogen burden, improving animal health, and offering viable alternatives to traditional antibiotics (Table 8).

### 5.2. Role in Aquaculture: Disease Control and Health Management

Antimicrobial peptides (AMPs) are increasingly recognized in aquaculture and biomedical applications due to their broad-spectrum activity, rapid response to infection, and cost-effective synthesis [240]. As highlighted by Ravichandran et al. [97], AMPs can be produced at relatively low cost, are stable during long-term storage, and are readily mobilized upon infection—making them valuable tools for prompt immune defense. Despite these advantages, several limitations may restrict their widespread application. For example, AMPs can exhibit reduced stability under varying pH conditions and may cause hemolytic effects due to their membrane-disruptive mechanisms [241,242]. Moreover, the industrial-scale production of AMPs remains challenging due to high technical complexity and associated costs [105]. Their efficacy may also be compromised in the presence of divalent ions such as Ca^2+^ and Fe^2+^ or serum components, potentially limiting their performance in vivo [243]. Additionally, the lack of comprehensive data on their toxicological effects, pharmacokinetics, and pharmacodynamics poses significant regulatory hurdles [244]. Climate change-induced ocean acidification may further impact AMP stability and activity in marine environments, though this remains an area requiring further study. Overall, while AMPs offer promising alternatives to conventional antibiotics, their practical application depends on addressing several biochemical, technical, and regulatory challenges.

#### 5.2.1. Antifungal Activities of AMPs

**Figure 6:**
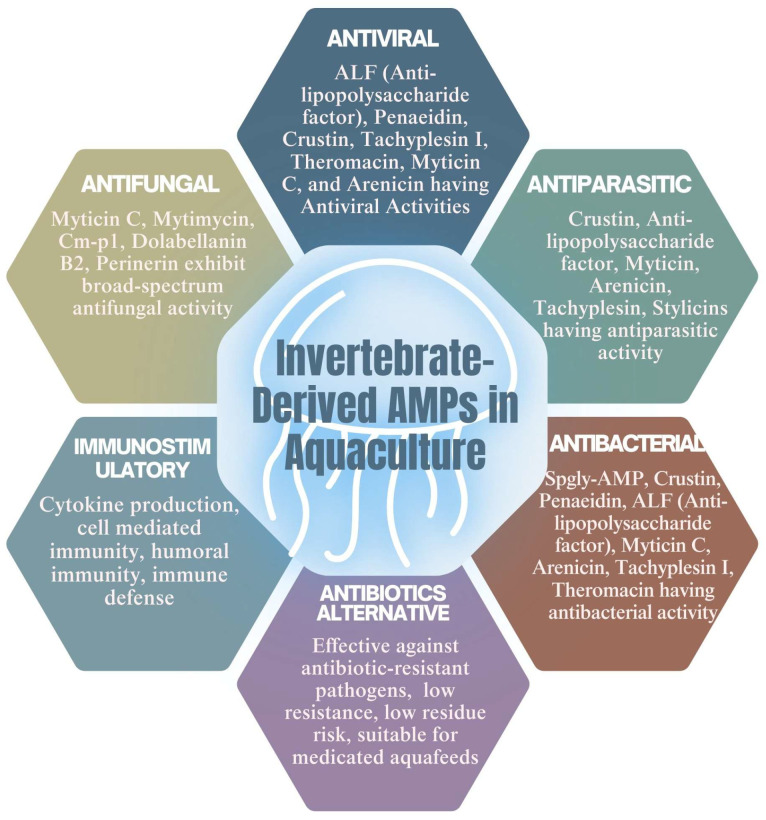
Potential application of AMPs in aquaculture.

AMPs derived from marine invertebrates are valuable tools for disease control in aquaculture systems (Figure 6). These peptides not only serve as key components of innate immunity but also function as potent suppressors of aquatic pathogens, particularly in environments with high microbial loads [240]. For instance, myticin C and mytimycin—cysteine-rich peptides from mussels—have demonstrated strong antifungal activity against Fusarium culmorum and Neurospora crassa, indicating their potential for managing fungal infections in shellfish hatcheries [211]. Similarly, perinerin, a broad-spectrum AMP from the clamworm (a marine annelid), has shown antimicrobial activity supportive of its use in benthic aquaculture environments [240].

In crustaceans, crustins such as carcinin—isolated from the shore crab Carcinus maenas—have demonstrated activity against Gram-positive bacteria, playing a critical role in host defense and survival in pathogen-rich aquatic systems [212]. Beyond their natural immune functions, these peptides hold translational promise in aquaculture. Strategies such as selective breeding for increased AMP expression or the direct application of AMPs in feed formulations could improve stock resilience, reduce antibiotic usage, and help mitigate the spread of antibiotic-resistant pathogens. Incorporating invertebrate-derived antifungal AMPs into hatchery and grow-out protocols offers a sustainable solution for disease prevention and health monitoring in aquaculture operations.

#### 5.2.2. The Antibacterial Activity of AMPs

Invertebrate-derived AMPs also exhibit significant antibacterial properties relevant to aquaculture health management (Figure 6). For example, dietary supplementation with 0.4% AMPs extracted from Pacific white-leg shrimp (*Litopenaeus vannamei*) resulted in improved growth, enhanced serum antioxidant levels, boosted innate immunity, and reduced mortality following *Vibrio* harveyi infection [245]. Similarly, Spgly-AMP peptides synthesized from the mud crab (*Scylla paramamosain*) demonstrated broad-spectrum antibacterial efficacy against both Gram-positive and Gram-negative bacteria [104]. While not invertebrate-derived, the AMP NKL-24 from zebrafish has shown cross-species immunomodulatory effects when administered to the Yesso scallop (*Patinopecten yessoensis*), reducing *Vibrio* parahaemolyticus infection and enhancing innate immune responses in the mollusk [111]. This cross-taxa effectiveness further supports the potential of AMP-based therapeutics across diverse aquaculture species.

Given the global rise in antibiotic resistance, AMPs offer a promising and sustainable alternative for managing infectious diseases in aquaculture. These naturally occurring molecules enhance host immunity and provide protection against a wide range of pathogens, including bacteria, fungi, viruses, and parasites. However, to fully realize their application in aquaculture, further research is needed—particularly under field conditions—to explore delivery methods, modes of action, and long-term safety. Continued investigation will be essential to successfully integrate AMPs into practical and scalable health management strategies in modern aquaculture.

### 5.3. Representative Case Studies of Marine Invertebrate AMPs

While marine invertebrate AMPs are broadly recognized for their antimicrobial and immunomodulatory roles, their translational potential becomes more apparent when considering specific examples. Here, we highlight three representative AMPs—Tachyplesin I, Myticin C, and Halocidin—that demonstrate both therapeutic promise and the key challenges that must be addressed for clinical or biotechnological application.

Tachyplesin I (horseshoe crab, *Tachypleus tridentatus*)

Tachyplesin I is a β-sheet antimicrobial peptide (AMP) first isolated from horseshoe crab hemocytes. It exhibits potent broad-spectrum activity against a wide range of pathogens, including multi-drug resistant (MDR) bacteria, though it also shows high hemolytic activity and cytotoxicity toward mammalian cells [246]. Notably, Tachyplesin I demonstrates efficacy against intracellular pathogens, such as uropathogenic *E. coli* (UPEC), a niche often inaccessible to conventional antibiotics [247]. Structural analogs, including [I11A] and [I11S] Tachyplesin I, retain strong antibacterial potency while markedly reducing cytotoxicity, thereby expanding the therapeutic window. Mechanistically, Tachyplesin I disrupts bacterial membranes and binds intracellular nucleic acids, impairing replication and gene expression. At sublethal concentrations, it also enhances host defenses by promoting zinc toxicity and stimulating macrophage responses [247].

Beyond antimicrobial activity, Tachyplesin I and its derivatives exhibit antitumor effects by inhibiting angiogenesis and inducing apoptosis in cancer cells [72]. Clinical application, however, is limited by hemolytic toxicity. Structural modifications such as amino acid substitutions and cyclization have been explored to overcome this limitation. While cyclization does not enhance antimicrobial or anticancer potency, it significantly improves serum stability and reduces hemolytic activity, broadening the therapeutic window [72,173]. Overall, Tachyplesin I represents a valuable scaffold for AMP-inspired drug design and remains a widely cited prototype for peptide-based anti-infective and anticancer therapeutics.

Myticin C (Mediterranean mussel, *Mytilus galloprovincialis*)

Myticin C, a cysteine-rich AMP expressed in mussel hemocytes, has emerged as a multifunctional effector extending well beyond classical antimicrobial defense. In vitro studies demonstrate its strong antiviral activity against the bivalve pathogen OsHV-1 in oysters and against human herpesviruses [173]. In addition, Myticin C functions as an immunomodulator, stimulating cytokine expression, enhancing hemocyte activity, and promoting hemocyte migration through its influence on gene expression [248]. It is constitutively stored in hemocyte vesicles and is rapidly upregulated upon injury, where it promotes tissue repair by activating hemocytes and directing their migration to damaged sites [95]. This dual role as both an antimicrobial effector and an immune regulator highlights potential applications not only in aquaculture for disease prevention but also in human medicine for immunomodulation. Recombinant expression systems have enabled scalable production of Myticin C, further supporting its translational promise. Although clinical trials are not yet available, the peptide’s broad bioactivity profile and feasibility of production position it as a strong candidate for future veterinary and biomedical applications.

Halocidin (tunicate, *Halocynthia aurantium*)

Halocidin, derived from the hemocytes of the tunicate *Halocynthia aurantium*, exhibits potent antifungal activity against Candida species, including strains associated with oral infections [249]. In addition to its antifungal properties, halocidin congeners (Khal) display strong antibacterial activity against major multidrug-resistant pathogens such as methicillin-resistant *Staphylococcus aureus* (MRSA), vancomycin-resistant Enterococcus (VRE), and *Pseudomonas aeruginosa*. Moreover, halocidin analogs have demonstrated protective efficacy in animal models of *Listeria monocytogenes* infection [250], highlighting their therapeutic promise as broad-spectrum antimicrobial agents. Mechanistically, Halocidin exerts a membranolytic effect on fungal pathogens while maintaining low cytotoxicity toward mammalian cells, a property that enhances its therapeutic potential [122]. In a murine model of oral candidiasis, a derivative peptide (HG1) significantly reduced fungal burden and demonstrated protective efficacy, underscoring its promise as a novel antifungal drug [251]. Current research efforts are directed toward improving stability and delivery, including encapsulation strategies to enhance bioavailability. Although foundational mechanistic studies date back to the early 2000s, recent reviews highlight Halocidin as one of the most translationally advanced marine invertebrate AMPs, particularly for antifungal therapy, thereby addressing a critical unmet need in clinical medicine [252].

Together, these examples illustrate the multifaceted therapeutic potential of marine invertebrate AMPs while underscoring key translational hurdles such as toxicity, stability, and production scalability. Tachyplesin I highlights the challenge of cytotoxicity and the importance of structural optimization; Myticin C demonstrates the value of multifunctionality and recombinant production; and Halocidin exemplifies the promise of marine AMPs in antifungal drug development. Collectively, they reinforce the view that marine invertebrate AMPs represent a rich and underexploited reservoir for next-generation therapeutics.

## 6. Artificial Intelligence in Antimicrobial Peptide Discovery, Design and Production

Artificial intelligence (AI) has emerged as a transformative force in the discovery, design, and production of antimicrobial peptides (AMPs), offering scalable, high-throughput alternatives to traditional homology-based and experimental screening methods while also enabling intelligent formulation and delivery optimization [115,253,254,255]. Conventional approaches typically rely on sequence similarity to known AMPs, which limits the discovery of structurally novel or functionally distinct candidates. In contrast, AI techniques, particularly deep learning (DL) and machine learning (ML), can detect complex sequence–function relationships and physicochemical patterns that may be invisible to rule-based systems [254,255]. These capabilities allow AI models to identify or design peptides with high antimicrobial potential, even in the absence of sequence homology to previously characterized AMPs [256].

Two principal AI-driven strategies dominate the field: AMP mining and AMP generation, both supported by discriminative and generative modeling techniques [257]. AMP mining utilizes discriminative classifiers such as support vector machines, random forests, and deep neural networks to scan natural or synthetic peptide sequences for antimicrobial activity while filtering out undesirable traits like toxicity or hemolysis [257]. Tools such as iAMP-2L, Deep-AmPEP30, and AMPScanner exemplify this approach by analyzing large, annotated datasets to predict peptide efficacy. Cutting-edge approaches like AMPGen, an evolutionary information-preserving diffusion model, have successfully generated target-specific AMPs with a synthesis success rate of ~95% (38 of 40 candidates) and demonstrated antibacterial activity in 81.6% of tested peptides [258]. Similarly, AMP-Designer, a foundation model-based system, has produced AMP candidates with strong in vitro efficacy and low hemotoxicity, achieving rapid design-to-validation cycles in under 48 days [258]. These strategies are often implemented within closed-loop design cycles, where generated sequences are filtered by predictive models, experimentally validated, and iteratively used to retrain the AI—creating a highly efficient discovery pipeline [258]. Several candidates identified through mining approaches have been experimentally validated, underscoring the practical utility of AI in real-world applications.

**Figure 7:**
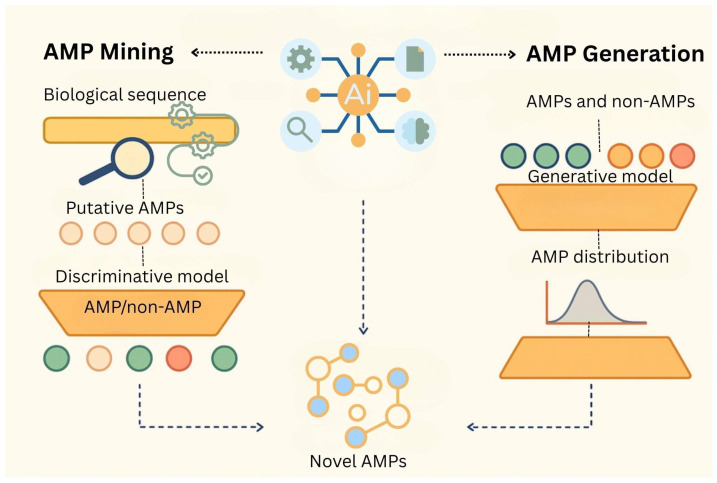
AI-driven AMP discovery workflow illustrates two major strategies: AMP mining and AMP generation. AMP mining involves analyzing biological sequences to identify putative AMPs, which are then classified using discriminative models to predict antimicrobial activity. In contrast, AMP generation leverages generative models trained on known AMPs and non-AMPs to design novel sequences optimized for antimicrobial properties. Both approaches aim to accelerate the identification of effective and diverse AMPs through machine learning tools.

In contrast, AMP generation involves the use of generative models including variational autoencoders (VAEs), generative adversarial networks (GANs), and large language models (LLMs) to create entirely new peptide sequences with optimized biological properties. These models are trained on curated AMP databases and are capable of preserving essential bioactive motifs while introducing novel variations to enhance potency and minimize toxicity. Advanced frameworks such as PepGAN and AMPGAN have demonstrated that synthetic peptides generated in silico can match or even outperform their natural counterparts. Typically, a closed-loop AMP design cycle is implemented, where generated sequences are filtered through predictive models, experimentally validated, and used to iteratively retrain and refine the algorithms—an approach that significantly accelerates discovery [259]. A visual summary of these AI strategies is illustrated in Figure 7, where AMP mining relies on scanning and classification, while AMP generation constructs de novo sequences using learned patterns from existing data. Together, these complementary approaches provide a robust pipeline for both identifying and engineering next-generation antimicrobial agents.

Beyond discovery and design, AI contributes directly to AMP production by guiding formulation strategies, optimizing delivery systems, and integrating with biomaterials and 3D printing technologies. AI also supports formulation and delivery optimization. Predictive algorithms can evaluate peptide–polymer compatibility to guide the design of nanocarriers, hydrogels, and nanoparticles that protect AMPs from degradation while enabling controlled release [78,260]. AI-guided biomaterial design has produced smart hydrogels incorporating AMPs, achieving >99.99% bactericidal activity and enhanced wound healing in vitro and in vivo [261]. Integration with 3D printing technologies expands possibilities for developing smart, stimuli-responsive biomedical devices with built-in antimicrobial functionality.

Despite its promise, AI-driven AMP discovery faces several ongoing challenges, including limited training datasets, model overfitting, interpretability issues, and the need for experimentally consistent benchmarking [115,262]. Generalizability across diverse pathogens and host environments also remains a key limitation. To address these issues, recent developments increasingly incorporate structural, chemical, and multi-omics data to enhance predictive accuracy and biological relevance. As algorithms continue to evolve and data quality improves, AI is expected not only to expedite AMP development but also to unlock peptide scaffolds with unprecedented therapeutic properties paving the way for innovative treatments in the era of rising antibiotic resistance.

## 7. Clinical Translation and Market Status of Marine Invertebrate AMPs

Despite the remarkable structural diversity and broad-spectrum antimicrobial activities of antimicrobial peptides (AMPs) from marine invertebrates, none have yet reached the pharmaceutical market as approved antimicrobial drugs. This gap reflects a combination of scientific, technological, and regulatory challenges. Marine invertebrate AMPs, while promising, are considered novel biomolecules, and thus require extensive preclinical and clinical evaluation before they can be considered for therapeutic application [263]. Preclinical studies assess pharmacokinetics, pharmacodynamics, and toxicity, while Phase I–III clinical trials are necessary to evaluate safety and efficacy in humans [264,265]. Moreover, robust study designs and post-marketing surveillance are essential to ensure long-term safety and therapeutic effectiveness.

Currently, the only FDA-approved marine invertebrate-derived peptide drug is Ziconotide (Prialt^®^), a synthetic analog of a conopeptide originally isolated from the cone snail *Conus magus* [265]. Ziconotide is approved for the management of severe chronic pain but does not possess antimicrobial activity [266]. This example demonstrates that marine invertebrate peptides can indeed be translated into clinically successful drugs, even though no antimicrobial peptide has yet followed this path.

In addition to peptides, other marine invertebrate-derived compounds have successfully entered the clinic. Cytarabine, a nucleoside analog originally isolated from the sponge *Cryptothethya crypta*, was approved by the FDA in 1969 under the brand name *Cytosar-U* for the treatment of leukemia, lymphoma, and acute myeloid leukemia (AML) [267]. Cytarabine works by blocking DNA synthesis, thereby disrupting cell replication. Similarly, Vidarabine, another nucleoside analog derived from the sponge *Tethya crypta*, acts as an antiviral drug by inhibiting viral DNA polymerase and has been used against herpes simplex virus (HSV) and varicella-zoster virus (VZV) [268]. Although these drugs are derived from marine invertebrates, they are nucleoside analogs rather than antimicrobial peptides, and their therapeutic activities (anticancer or antiviral) are distinct from those of AMPs. Nevertheless, their success highlights the feasibility of translating marine-derived bioactive molecules into clinically approved drugs.

At present, several marine invertebrate-derived AMPs such as Tachyplesin I, Myticin, Halocidin, Pardaxin, and Cm-p5 are under active investigation. These peptides have demonstrated potent antimicrobial activities against multidrug-resistant bacteria and fungi in preclinical studies, yet they remain in the research stage and have not advanced to clinical approval [4,263,264].

**Figure 8:**
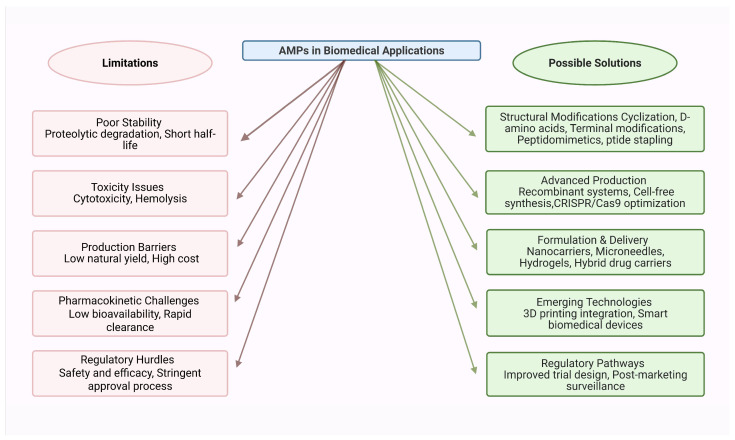
Limitations and solutions for marine invertebrate AMP-based therapeutics.

The absence of marketed antimicrobial drugs derived from marine invertebrate AMPs can be attributed to several challenges (Figure 8). First, many AMPs exhibit poor stability in physiological environments due to rapid proteolytic degradation, which reduces systemic bioavailability [269]. Second, cytotoxicity and hemolytic effects at therapeutic concentrations have limited their clinical development for human use [114]. Third, large-scale production remains a major hurdle; natural extraction yields very low amounts of peptides, while recombinant or chemical synthesis often incurs high costs and technical bottlenecks [270]. Finally, stringent regulatory requirements for demonstrating safety, efficacy, and pharmacokinetics of peptide drugs add additional barriers to clinical translation [264].

To overcome these limitations, multiple strategies are being pursued (Figure 8). Structural modifications such as cyclization, incorporation of D-amino acids, terminal modifications (e.g., acetylation, amidation), and peptide stapling can increase proteolytic stability, reduce cytotoxicity, and improve pharmacological properties. Sequence modifications and peptidomimetics further enhance drug-like characteristics. On the production side, advances in recombinant systems, including engineered microorganisms and cell-free protein synthesis platforms, are providing scalable and cost-effective routes for AMP manufacturing. Cell-free systems, in particular, are advantageous for producing AMPs that are otherwise difficult to express due to toxicity or instability [271].

Formulation and delivery technologies also play an important role in clinical translation. Nanocarriers, microneedles, and hydrogels can encapsulate AMPs, protecting them from enzymatic degradation and enabling controlled, sustained release at the target site. Hybrid drug carriers and smart delivery systems are being developed to further enhance AMP stability, reduce side effects, and improve tissue penetration [269]. Cutting-edge technologies such as CRISPR/Cas9-mediated sequence optimization are also being explored to fine-tune AMP properties for enhanced potency and reduced toxicity [272]. Furthermore, integration of AMPs with 3D printing technologies is enabling the creation of smart biomedical devices and personalized therapeutic platforms, although cost-effectiveness and scalability remain challenges for widespread adoption [273].

In summary, no marine invertebrate AMP has yet been approved for antimicrobial therapy. However, the success of Ziconotide as a marine invertebrate peptide drug provides proof of concept for clinical translation. The historical examples of Cytarabine and Vidarabine illustrate that marine invertebrate-derived compounds can overcome regulatory hurdles and achieve market approval, even if they are not AMPs. While challenges such as instability, cytotoxicity, and production bottlenecks continue to slow progress, rapid advances in peptide engineering, recombinant production, and drug delivery systems are paving the way for marine AMPs to emerge as next-generation anti-infective agents in the clinic.

## 8. Conclusions

Marine-derived antimicrobial peptides (AMPs) represent a vast and largely untapped source of biologically potent molecules with significant therapeutic and biotechnological potential. Produced by diverse marine invertebrates, these structurally varied peptides play vital roles in innate immunity and exhibit broad-spectrum activities against bacteria, fungi, viruses, parasites, and even cancer cells. Their exceptional physicochemical stability, adapted to harsh marine environments, further highlights their promise as natural immune effectors and valuable templates for next-generation drug development. Marine AMPs offer considerable benefits for human health, veterinary medicine, and aquaculture, including antimicrobial efficacy, immune enhancement, and growth promotion, making them attractive alternatives amid rising antimicrobial resistance. Recent advances in high-throughput sequencing, omics technologies, and particularly artificial intelligence (AI) have transformed AMP discovery and design. AI-driven approaches facilitate rapid identification, optimization, and generation of novel peptides with enhanced bioactivity and reduced toxicity, significantly accelerating discovery while lowering costs. These innovations provide scalable solutions to the urgent global demand for new antimicrobials.

Nevertheless, challenges remain. Many marine species lack comprehensive genomic data, and a majority of AMPs have yet to be functionally characterized. Large-scale production methods are still limited, and there is insufficient understanding of AMP pharmacokinetics, toxicology, and the potential for resistance development. Moreover, the efficacy and safety of marine AMPs require further validation under physiological and field conditions. Future research should prioritize the exploration of underrepresented marine taxa using integrated omics to uncover novel AMPs. In-depth mechanistic studies, supported by advanced structural biology techniques, are needed to elucidate AMP actions and interactions, including synergy with other antimicrobials. Developing cost-effective, scalable production platforms is essential for practical applications. Continued refinement of AI-based discovery tools, leveraging diverse datasets, will enable the design of peptides tailored to specific clinical and aquaculture needs. Finally, translating these discoveries into real-world use demands rigorous preclinical and clinical validation, alongside careful assessment of immunostimulatory potential, environmental impact, and resistance risks to ensure sustainable and effective deployment.

## Figures and Tables

**Table 1:** Representative antimicrobial peptides (AMPs) from aquatic annelids.

Group	Peptide Name	Source Organism	Structure/Class	Key Features	Antimicrobial Activity	Reference(s)
Clitellata	Lumbricin	*Lumbricus rubellus*, *Hirudo medicinalis*	Proline-rich	Immune response, CNS regeneration	Broad-spectrum (bacteria); *D. nishinomiyaensis*	[43,44,45,46]
Polychaeta	Theromyzin	*Theromyzon tessulatum*	Anionic, α-helical	First anionic AMP in invertebrates	Not specified	[47]
	Arenicin	*Arenicola marina*	β-hairpin, disulfide-stabilized	Extremophile; synthetic analogs developed	Broad-spectrum (bacteria)	[48,49,50]
	Abarenicin	*Abarenicola pacifica*	BRICHOS-related	Extremophile; BRICHOS domain	Not specified	[51]
	UuBRI-21	*Urechis unicinctus*	BRICHOS-related	β-sheet; thermal adaptation	Not specified	[51]
	Nicomicin	*Nicomache minor*	α-helical	Anticancer activity	Antibacterial, anticancer	[52]
	Alvinellacin	*Alvinella pompejana*	β-sheet, disulfide-stabilized	Deep-sea AMP; heat-tolerant species	Antibacterial	[53,54]
	Capitellacin	*Capitella teleta*	β-sheet, disulfide-stabilized	Homolog of alvinellacin	Antibacterial	[54]
	Polaricin	*Amphitritides* sp.	Not specified	Antarctic AMP	Antibacterial	[55]
	HfBRI-25/28	*Heteromastus filiformis*	β-hairpin (HfBRI-25), α-helical (HfBRI-28)	Polar origin; disulfide-stabilized	Not specified	[55,56,57]
	AmBRI-44a	*Arenicola marina*	Defensin-like, disulfide-stabilized	Four disulfide bridges	Not specified	[57]
	Hedistin	*Hediste diversicolor*	Cationic α-helical	Brominated tryptophan residues	Not specified	[39]
	Perinerin	*Perinereis aibuhitensis*	Cationic α-helical	Two possible intramolecular disulfide bridges	Not specified	[58]

**Table 3:** Antimicrobial peptides (AMPs) identified from Cephalochordata and Tunicata.

Group	Peptide Name	Source Organism	Structure/Class	Key Features	Antimicrobial Activity	Reference(s)
Cephalochordata	BjAMP1	*Branchiostoma japonicum*	Two α-helices connected by a reverse turn (amphipathic)	Penetrates membranes without disrupting structure; binds LPS/LTA; may bind DNA/RNA; non-toxic to mammalian cells	Broad-spectrum antibacterial activity	[112,113,114,115,116]
	mBjAMP1 (analogs)	Synthetic analogs	Modified α-helical peptides	Enhanced antimicrobial and antibiofilm activity through amino acid modifications	Improved broad-spectrum activity	[115,117]
Tunicata	Styelins	*Styela clava*	Phenylalanine-rich	Identified from ascidians	Active against Gram-positive, Gram-negative bacteria, and fungi	[118,119,120]
	Clavanins	*Styela clava*	α-helical	Histidine-rich variants	Broad-spectrum antimicrobial activity	[118,119,120]
	Clavaspirin	*Styela clava*	Histidine-rich	Functions not fully described	Broad-spectrum antimicrobial activity	[118,119,120]
	Halocidin	*Halocynthia aurantium*	Two amphipathic α-helices with disulfide bond	Two monomers (18 and 15 amino acids); 18-residue monomer more active; synthetic analogs (e.g., di-K19Hc) show improved activity	Broad-spectrum antibacterial activity	[39,47,53,121,122,123]
	Dicynthaurin	*Halocynthia aurantium*	Dimer of two 30-aa amphipathic α-helical peptides	Linked by single cysteine disulfide bond; α-helical conformation	Broad-spectrum antibacterial activity	[121,123]
	Halocyntin, Papillosin	*Halocynthia papillosa*	Not detailed	Identified from hemocytes	Broad-spectrum antibacterial activity	[124]
	Ci-MAM-A24, Ci-PAP-A22	*Ciona intestinalis*	Not specified (from EST database)	Early AMP identification from EST screening	Antibacterial activity	[125,126,127]
	P-02 to P-10	*Ciona intestinalis*	Short ORF-derived peptides	Predicted from sORFs; 5 out of 10 tested peptides showed activity	Antibacterial activity	[128]
	Turgencins (AMox1, etc.)	*Synoicum turgens*	Cysteine-rich, unusual disulfide bridges, amidated C-end	Methionine oxidation variants; AMox1 most potent; active against melanoma and fibroblast cells	Broad-spectrum antibacterial and anticancer activity	[129]
	StAMP-1 to StAMP-11	Synthetic (based on turgencin)	Synthetic analogs	StAMP-9 most potent; selective antimicrobial with low hemolysis and cytotoxicity	Broad-spectrum antibacterial, antifungal, and non-cytotoxic	[129]

**Table 4:** Antimicrobial peptides (AMPs) identified in cnidarians.

Group	Peptide Name	Source Organism	Structure/Class	Key Features	Antimicrobial Activity	Reference(s)
Anthozoa	Damicornin	*Pocillopora damicornis*	Cysteine-rich (6 cysteines)	Gene expression repressed by *Vibrio coralliilyticus*	Gram-positive bacteria, *Fusarium oxysporum* (fungus)	[137]
	AmAMP1	*Acropora millepora*	Cysteine-rich (6 cysteines)	Expressed in ectodermal cells during coral development	Broad-spectrum: Gram-positive and Gram-negative bacteria	[138]
	Pd-AMP1	*Phyllogorgia dilatata*	β-hairpin structure	Soft coral-derived peptide	Gram-positive bacteria	[139]
	Crassicorin	*Urticina crassicornis*	Double β-hairpin, 6 cysteines	Sea anemone-derived, structurally stable	Antibacterial activity	[140]
	Equinins	*Actinia equina*	Not specified	No hemolysis on human cells; low antibacterial activity	Weak antibacterial activity	[141]
Medusozoa	Aurelin	*Aurelia aurita*	6 cysteines; 2 helices + coil	Isolated from jellyfish mesoglea	Antibacterial activity	[142,143]
	Arminin 1a-C	*Hydra*	α-helical peptide	Selective anticancer effects; no hemolysis; C-terminal domain of Arminin 1a	Strong antibacterial; anti-leukemia cell viability	[144,145]
	Periculin-1	*Hydra*	Anionic N-term, cationic C-term; 8 cysteines	Expressed in female germline; controls bacterial colonization during embryogenesis	Potent antimicrobial activity	[146,147]

**Table 5:** Antimicrobial peptides (AMPs) identified from Echinodermata.

Group	Peptide Name	Source Organism	Structure/Class	Key Features	Antimicrobial Activity	Reference(s)
Asteroidea	PpCrAMP	*Patiria pectinifera*	β-hairpin	Contains two β-strands linked by a random coil	Antibacterial activity	[153]
Echinoidea	Strongylocins	*Strongylocentrotus droebachiensis*, *S. purpuratus*, *Echinus esculentus*	Cysteine-rich, cationic peptide	Six-cysteine motif; brominated tryptophan	Strong antibacterial activity	[154,155,156]
Echinoidea	Centrocins	*Strongylocentrotus droebachiensis*, *Echinus esculentus*	Heterodimeric peptide	30-residue heavy chain + 12-residue light chain linked by disulfide bond; brominated tryptophan	Strong antibacterial and some antifungal activity	[154,155,156]
Holothuroidea	Holothuroidins	*Holothuria tubulosa*	Cationic α-helical peptide	Small peptides; weak antibacterial potency but notable antibiofilm properties	Weak antibacterial, antibiofilm activity	[157,158]

**Table 6:** Representative Antimicrobial Peptides (AMPs) Identified in Mollusca.

Group	Peptide Name	Source Organism	Structure/Class	Key Features	Antimicrobial Activity	Reference(s)
Bivalvia	Mytilins	*Mytilus* spp.	Cysteine-rich	Signal peptide + mature region + C-terminal extension	Bacteria, fungi	[160,171]
	Mytimycins	*Mytilus* spp.	Cysteine-rich	Similar structure; precursor-based	Bacteria, fungi	[94,160,172]
	Myticins	*Mytilus* spp.	Cysteine-rich	Precursor-derived; conserved motif	Bacteria, fungi, viruses	[73,95,173]
	Myticusins	*Mytilus* spp.	Cysteine-rich	Target parasites in addition to microbes	Bacteria, fungi, parasites	[161,174,175]
	Myticalins	*Mytilus* spp.	Cationic, α-helical + random coil	Broad-spectrum, gene-encoded	Gram-positive and Gram-negative bacteria	[162,176,177]
	Mytichitins	*Mytilus* spp.	Chitin-binding domain	Targets chitin; enhances antimicrobial defense	Bacteria, fungi, parasites	[81]
	Ap	*Argopecten purpuratus*	Proline-rich, cationic	Non-mussel species; strong antifungal effect	Fungi	[178]
	Cg-Prp	*Crassostrea gigas*	Proline-rich	Limited direct effect; synergistic with Cg-Def	Synergistic enhancement with defensin	[102,179]
	URP20	*Crassostrea hongkongensis*	α-helical, cationic	Potent activity; non-toxic to mammalian cells	Bacteria, fungi	[163]
	Molluscidin	Crassostrea gigas, Atrina pectinata, Haliotis discus	Non-amphipathic; alternating α-helix and random coil	Contains repeated dibasic residues; isolated from gill tissue; low cytotoxicity	Active against Gram-positive and Gram-negative bacteria	[106,180]
Cephalopoda	Octopartenopin	*Octopus vulgaris*	Random coil, pentapeptide	Derived from sucker tissue	Antibacterial	[164]
	Octominins	*Octopus minor*	Cationic, α-helical	Well-characterized peptide family	Antibacterial	[165,166]
	Octopromycin	*Octopus minor*	Cationic, α-helical	Novel structure	Antibacterial	[99]
	Octoprohibitin	*Octopus minor*	Cationic, α-helical	Immunomodulatory potential	Antibacterial	[181]
	KT19, VA20, GR21	*Sepia officinalis*	Putative peptides	Identified via peptide screening	Antimicrobial potential	[182]
	NF19, AV19, GK28	*Sepia officinalis*	Putative peptides	Active peptide variants	Antimicrobial potential	[183]
Gastropoda	Cm-p1	*Cenchritis muricatus*	α-helical	Antifungal without mammalian toxicity	Fungi	[167,168]
	Peptide 4	*Rapana venosa*	Random coil, proline-rich	Invasive species; Gram-negative targeting	Gram-negative bacteria	[184]
	Peptide 7	*Rapana venosa*	α-helical	Broad-spectrum potential	Gram-negative bacteria	[184]
	Bb-AMP4	*Filopaludina bengalensis*	Not defined	Freshwater snail origin	Gram-positive bacteria	[185]
	Pom-1 (Closticin 574)	*Pomacea poeyana*	α-helical	Antiviral and antibacterial activity	Bacteria, viruses (e.g., ZIKV)	[169]
	Pom-2 (Cecropin D-like)	*Pomacea poeyana*	α-helical	Narrower spectrum	Antibacterial	[169]
	Dolabellanin B2	*Dolabella auricularia*, *Peronia peronii*	α-helical	Found in sea hare and sea slug	Antibacterial, antifungal	[170,186]

**Table 7:** Bioactive peptides derived from marine Porifera.

Source	Peptides	Activity	References
Porifera peptides	Callyaerin A and B	Antimicrobial	[203]
	Theonellamide F	Antimicrobial	[204]
	Theonellamide G	Antimicrobial	[204]
	Koshikamides F and H	Antiviral	[79]
	Celebesides A–C	Antiviral	[79]
	Mirabamides A–D	Antiviral	[205]
	Mirabamides E–H	Antiviral	[205]
	Stellettapeptins A and B	Antiviral	[205]
	Barettin and 8,9-dihydrobarettin	Antifouling	[206]
	Barrettides A and B	Antifouling	[207]

**Table 8:** Therapeutic Applications of Lactoferrin-Derived Peptides in Veterinary Medicine.

Animal Model	Condition	Peptide Used	Pathogen(s)	Delivery Method	Key Outcomes	Reference(s)
Dairy cattle (cow)	Bovine mastitis	Lf + Penicillin G	*Staphylococcus aureus* (β-lactam-resistant)	Intramammary infusion	Cure rate improved from 12.5% to 33%; β-lactamase activity reduced	[231]
Dairy cattle (cow)	Subclinical mastitis	Lf Hydrolysate (LFH)	*E. coli*, *Staphylococcus* spp.	Intramammary infusion	Bacterial load reduced on Day 1; full recovery in 14 days	[232]
Dairy cattle (cow)	Protothecal/fungal mastitis	bLfcin	*Prototheca zopfii*, yeast spp.	In vitro	Demonstrated antimicrobial activity (in vitro only)	[233]
Goat	Preventive udder infection	bLfcin	*S. aureus*, *E. coli*	Recombinant plasmid vector	Peptide persisted in milk up to 6 days; milk showed antibacterial activity	[234]
Piglets	Enterotoxigenic *E. coli*	cipB-Lfcin	*E. coli*	Dietary supplementation (100 mg/kg)	Improved gut morphology, lower cytokine levels, reduced E. coli count	[235]
Piglets	Enterotoxigenic *E. coli*	cipB-Lfcin-Lframpin	*E. coli*	Dietary supplementation (100 mg/kg)	Enhanced growth, gut health similar to colistin treatment	[236]
Piglets	Enterotoxigenic *E. coli*	bLfcin/Lfampin	*E. coli*	Recombinant yeast (*P. pastoris*)	Improved growth and gut health	[237]
Piglets	Enterotoxigenic *E. coli* F4	bLfcin	*E. coli* F4	Oral administration	Reduced diarrhea and maintained healthy weight; no toxicity	[238]
Dogs	Fungal and bacterial otitis	bLfcin + verbascoside	*Malassezia* spp., others	Topical emulsion	Reduced microbial load, clinical improvement in 7 days	[239]

Lf—Lactoferrin; bLfcin—Bovine Lactoferricin; Lfampin—Lactoferrampin; LFH—Lactoferrin Hydrolysate; cipB-Lfcin—Fusion protein combining cipB signal peptide with Lfcin for targeted stomach release; cipB-Lfcin- Lframpin—Dual-function fusion peptide of Lfcin and Lframpin linked with cipB; *P. pastoris*—Pichia pastoris (recombinant yeast expression system); F4—Enterotoxigenic *Escherichia coli* F4 strain.

## Data Availability

No datasets were generated or analyzed during the current study.
